# The Transcriptome of Equine Peripheral Blood Mononuclear Cells

**DOI:** 10.1371/journal.pone.0122011

**Published:** 2015-03-19

**Authors:** Alicja Pacholewska, Michaela Drögemüller, Jolanta Klukowska-Rötzler, Simone Lanz, Eman Hamza, Emmanouil T. Dermitzakis, Eliane Marti, Vincent Gerber, Tosso Leeb, Vidhya Jagannathan

**Affiliations:** 1 Swiss Institute of Equine Medicine, Vetsuisse Faculty, University of Bern and Agroscope, Bern, Switzerland; 2 Institute of Genetics, Vetsuisse Faculty, University of Bern, Bern, Switzerland; 3 Division of Pediatric Hematology/Oncology, Department of Pediatrics, Bern University Hospital, Bern, Switzerland; 4 Clinical Immunology Group, Department of Clinical Research and Veterinary Public Health, University of Bern, Bern, Switzerland; 5 Department of Genetic Medicine and Development, University of Geneva Medical School, Geneva, Switzerland; 6 Institute of Genetics and Genomics in Geneva, Swiss Institute of Bioinformatics, Geneva, Switzerland; University of North Carolina at Charlotte, UNITED STATES

## Abstract

Complete transcriptomic data at high resolution are available only for a few model organisms with medical importance. The gene structures of non-model organisms are mostly computationally predicted based on comparative genomics with other species. As a result, more than half of the horse gene models are known only by projection. Experimental data supporting these gene models are scarce. Moreover, most of the annotated equine genes are single-transcript genes. Utilizing RNA sequencing (RNA-seq) the experimental validation of predicted transcriptomes has become accessible at reasonable costs. To improve the horse genome annotation we performed RNA-seq on 561 samples of peripheral blood mononuclear cells (PBMCs) derived from 85 Warmblood horses. The mapped sequencing reads were used to build a new transcriptome assembly. The new assembly revealed many alternative isoforms associated to known genes or to those predicted by the Ensembl and/or Gnomon pipelines. We also identified 7,531 transcripts not associated with any horse gene annotated in public databases. Of these, 3,280 transcripts did not have a homologous match to any sequence deposited in the NCBI EST database suggesting horse specificity. The unknown transcripts were categorized as coding and noncoding based on predicted coding potential scores. Among them 230 transcripts had high coding potential score, at least 2 exons, and an open reading frame of at least 300 nt. We experimentally validated 9 new equine coding transcripts using RT-PCR and Sanger sequencing. Our results provide valuable detailed information on many transcripts yet to be annotated in the horse genome.

## Introduction

Morphology studies have shown that equine and human lungs bear a striking resemblance [1,2]. In temperate climates 10% to 20% of stabled horses succumb to a condition called recurrent airway obstruction (RAO, also known as heaves) characterized by stable dust-induced inflammation, bronchospasm and airway remodeling. These characteristics are very similar to human asthma. RAO is a naturally occurring disease, showing cumulative effects of multiple episodes of dust-induced exacerbation in a context of complex gene and environment interactions [3,4]. Another interesting characteristic is the highly sensitive response of horses to lipopolysaccharides (LPS), which is also similar to humans [5].

There is however limited information on the transcriptome profile of lung tissue and immune cells in horses. This limitation comes from the fact that there is only a small number of expressed sequence tags and cDNA data for horses deposited in public databases (37,756 entries in NCBI dbEST, release 130101). Hence, the current gene models are derived based on a combination of *ab initio* methods, homology based studies, similarity and motif analysis programs. This is currently rapidly changing with several groups publishing digital gene analyses from a variety of horse tissues, including muscle, leukocytes, cartilage, brain, reproductive tissue, embryos, sperm, and blood [6–16]. These studies have catalogued several non-coding genes in addition to protein coding gene isoforms, and structural annotations of existing genes.

The RNA-seq technology facilitates comprehensive whole transcriptome analyses without previous knowledge of the transcriptome structure [17,18]. Short sequencing reads from cDNA are either first mapped to the reference genome sequence or, in the absence of a reference genome, *de novo* assembled into transcript contigs [19–22]. This methodology also allows to annotate non-coding genes and to find alternatively spliced isoforms for each gene locus [10,21,23–25]. RNA-seq is particularly meaningful in studies of non-model organisms with poor genome annotation. This technology enables the identification of new, and sometimes even species-specific, transcripts. Using RNA-seq 13,086 unannotated transcripts (33% of all transcripts identified in the study) were identified in bovine skin [24]. Park et al. identified 20,428 novel transcripts (60% of all transcripts identified in the study) expressed in equine muscle and blood samples [6].

The equine gene set annotated by the Ensembl pipeline (build 72.2) contains 20,449 protein-coding genes and 4,400 pseudogenes. Despite the fact that the number of protein coding genes is similar to human, there are only 1,635 equine protein coding genes (8%) with more than one transcript annotated. In contrast, in human 18,516 (82%) from the 22,680 protein coding genes have more than one transcript annotated. Therefore, any isoform-specific gene expression analysis of the horse transcriptome, using the currently available set of annotated transcripts, will be based on a highly incomplete annotation. Although it is computationally more challenging, the expression analysis at the isoform level has been shown to be more accurate in human gene expression studies [21,26–31].

To facilitate meaningful future global gene expression studies in horses we performed an RNA-seq experiment with the goal of improving the structural annotation of the transcriptome of peripheral blood mononuclear cells (PBMCs). PBMCs consist of cells involved in both innate and adaptive immune response. Changes in the population of PBMCs after antigen stimulation were reported in 1968 in humans and even earlier in animals [32]. The traffic of immune cells in the blood during infection indicates the type of infection independent of its specific localization. Therefore, PBMCs are a common target of immunological studies.

Our analysis provides a very comprehensive snapshot of the equine PBMC transcriptome. Moreover, this study highlights the value of RNA-seq in identifying novel genes and isoforms that underlie the immune response to allergens.

## Materials and Methods

### Ethics statement

All animal experiments were performed according to the local regulations. The horses in this study were examined with the consent of their owners. This study was fully approved by the Ethical Committee of the Canton of Bern (BE33/07, BE58/10 and BE10/13).

### Samples

We collected blood samples from 85 Warmblood horses (stallions: n = 20, mares: n = 36, geldings: n = 29). Among the horses, 40 were diagnosed with RAO, and 45 were RAO-non-affected. The samples were collected as described [33]. All of the RAO-affected horses were in remission phase at the time of sample collection. PBMCs were isolated by density gradient centrifugation [34]. Approx. 8 x 10^6^ PBMCs were then cultured in 4 ml medium for 24 hours with different stimulating factors [33]. For this study, we used samples stimulated with lipopolysaccharides (LPS of *E*. *coli*, Sigma–Aldrich) at a concentration of 250ng/ml (in the unrelated group of horses 12 samples were stimulated with LPS at higher concentration: 5 or 10 μg/ml, and two samples were stimulated with LPS at lower concentration: 80 pg/ml); hay dust extract (HDE) [33,35] at three concentrations: 12, 9 or 6 μg/ml; or recombinant cyathostomin antigen (RCA) [36] at two concentrations: 4 or 1 μg/ml. As a reference, PBMCs were cultured under the same conditions, but without stimulating factor (mock). In total, we studied 42 groups of samples with 6 to 29 biological replicates per group. The exact number of replicates is given in S1 Table.

### RNA isolation and sequencing

RNA was isolated from cultured cells as described in Lanz et al. [33]. We measured the quality and quantity of the isolated RNA with an Agilent 2100 Bioanalyzer (Agilent Technologies) and Qubit 2.0 Fluorometer (Life Technologies). Approximately 500 ng of high quality RNA (RNA integrity number: RIN > 8) was used for non-directional paired-end RNA library preparation (TruSeq Sample Preparation Kit v2 guide Part #15026495 Rev.D, Illumina).

Total mRNA libraries were randomly multiplexed in 12 samples per lane and sequenced on the Illumina HiSeq2000 platform using 2 x 50 bp paired-end sequencing cycles. The Illumina BCL output files with base calls and qualities were converted into FASTQ file format and demultiplexed with the casava (v1.8.2) software.

### Quality control of reads

The reads in FASTQ format were processed for quality checks using the FastQC tool (v0.10.1; http://www.bioinformatics.babraham.ac.uk). The statistics on the number of reads per library, base quality scores, read length, GC content, number of missing base calls, and number of unique 15-mers was collected separately for the forward and reverse reads in each of the lanes.

### Mapping

Good quality reads were then mapped to the most recent horse reference genome assembly, EquCab2 [37], using the GEM mapper (v1.6.2) [38] guided by existing annotations from Ensembl (release 72). For the GEM mapper (v1.6.2) we allowed for a maximum of two mismatches and a maximum intron length of 500 kbp. For all other parameters we kept the default values. The guided procedure maps reads to existing gene regions. The procedure also allows for mapping sequence reads to transcript regions not yet annotated in the reference genome. Mapping quality control was performed using the RSeQC package [39]. The saturation status of splicing junction detection, coverage uniformity over gene body, splice junction coverage of known gene bodies, read distribution in the genome, and the origin (known/novel) of splice sites were tested. The binary alignment files (BAMs) are available from the European Nucleotide Archive (ENA) database (http://www.ebi.ac.uk/ena/data/view/PRJEB7497).

### Transcriptome assembly

We assembled the reference based transcriptome for each sample using Cufflinks (v2.1.1) [21,40]. All 561 assemblies were then merged into one transcriptome assembly, guided by reference gene structures, using Cuffmerge (v2.1.1) [40]. Reference transcripts not expressed in any of the samples studied were discarded from the assembly.

### Expression quantification

The merged file was used as a reference for the Cuffquant and Cuffnorm tools (Cufflinks v2.2.0) for the transcript abundance estimation [40]. We estimated the median of fragment per kilobase per million fragments mapped (FPKM) for each transcript. Transcripts with expression values less than 0.01 FPKM per sample were considered as lowly expressed transcripts and excluded from further analysis.

### Identification of unknown transcripts

The merged assembly was compared with the reference gene models predicted by either the Ensembl or the NCBI pipelines on the EquCab2 genome assembly, available in public databases. Subsequently, we eliminated all the transcripts that corresponded to known/predicted models. The final reduced data set contained only transcripts with class code “u”, according to Cuffcompare, and we refer to them from here on as unknown transcripts.

### Classification of unknown transcripts

We obtained FASTA formatted sequences from the horse reference genome for each unknown transcript using the gffread function of Cufflinks (v2.1.1). We then performed sequence homology searches against the expressed sequence tags database (dbEST, NCBI, release 130101) [41] using the BLASTN algorithm (v2.2.26+, e-value threshold = 1e-5) [42].

All unknown transcripts were then evaluated for coding potential using the Coding Potential Calculator (CPC) [43]. The CPC tool uses support vector machines to calculate the coding potential of a transcript. It takes e.g. open reading frame (ORF) length, sequence conservation, and alignment information into consideration for the prediction. It estimates the coding potential scores: negative for non-coding, positive for coding transcripts. According to the program’s documentation, transcripts with a score between zero and one have weak coding potential. All transcripts with positive coding score and without match in the EST database were considered as potentially new horse-specific protein coding transcripts.

### Validation of potentially coding transcripts

Potentially coding transcripts with at least two exons, an ORF ≥ 300 bp, and mean expression across samples ≥ 1 FPKM were selected for experimental validation by reverse transcription polymerase chain reaction (RT-PCR). RNA samples for experimental confirmation were reverse transcribed into cDNA using SuperScript II reverse transcriptase (Invitrogen) and oligo(dT) primers. The cDNA was then used for PCR amplification with specific primers designed with Primer3Web (v4.0.0.0) [44,45] and AmpliTaq Gold 360 Mastermix (Applied Biosystems). The PCR steps were as follows: initial denaturation (95°C for 10 min); 36 cycles of denaturation (95°C for 30 s), annealing (58°C for 30 s), and extension (72°C for 40 s); and final extension (72°C for 7 min). RT-PCR products were directly sequenced on an ABI 3730 capillary sequencer (Applied Biosystems) after treatment with exonuclease I (New England Biolabs) and rAPid Alkaline Phosphatase (Roche). We analyzed the Sanger sequence data with Sequencher 5.1 (GeneCodes).

## Results

### Sequencing and mapping

In this study, we sequenced 561 RNA libraries derived from *in vitro* stimulated equine PBMCs. We obtained between 8,590,084 and 141,367,074 paired-end reads per library (17 million paired-end reads per library on average, S1 Fig.). In total, our dataset consisted of 19.33 billion (10^9^) reads of length 49 bp. The quality of the reads was high–the mean base quality score in the Phred scale [46,47] was 35.68, indicating that the base call accuracy was above 99.97%. The reads were characterized by high variation in the sequence content, and there were no indications of high level of duplication or GC-bias (on average almost 10^7^ unique 15-mers and 49.35% GC content per sample).

**Fig 1:**
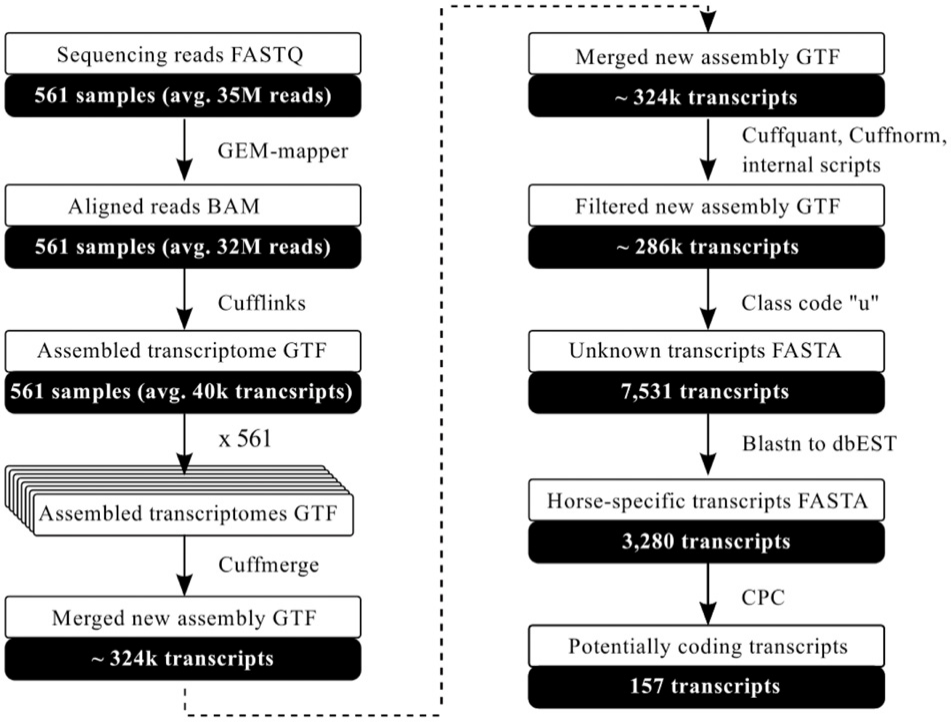
Workflow of the analysis. The principal steps of the analysis and the format of the output files are given.

The reads were mapped to the EquCab2 reference genome with the GEM mapper. The efficiency of the mapping was high and reached 93% with 18.16 x 10^9^ mapped reads and 17.09 x 10^9^ uniquely mapped reads. Less than half of the reads (n = 7.8 x 10^9^; 40%) covered known exons of 22,229 genes from a total of 26,991 genes annotated in Ensembl (Ensembl, release 72) (S1 Fig.). Of the 22,229 genes explained by at least one read in our dataset, 20,617 (93%) are currently annotated as single-transcript genes by Ensembl. The mapped reads were used for generating a new transcriptome for PBMCs. The principal steps of the analysis are shown in Fig. 1.

### Transcriptome assembly

On average, one individual RNA-seq assembly consisted of 39,921 transcripts belonging to 34,132 genes. The individual RNA-seq assemblies contained 35% single-exon transcripts on average. We then merged the 561 individual transcript assemblies into one merged assembly. Due to the low proportion of reads spanning the Ensembl gene structures, the merge was guided by annotations from Ensembl (Ensembl, release 72) and NCBI Gnomon (NCBI, release 101).

After merging the 561 assemblies and removing non-expressed reference sequences, the merged assembly included 316,457 transcripts expressed from 42,615 gene loci. The number of single-transcript genes was 17,136 (40%). We discovered 8,483 new gene loci from the merged assembly. Two thirds of the single-transcript genes (n = 11,802; 69%) were associated with equine genes already annotated in Ensembl or NCBI databases.

**Fig 2:**
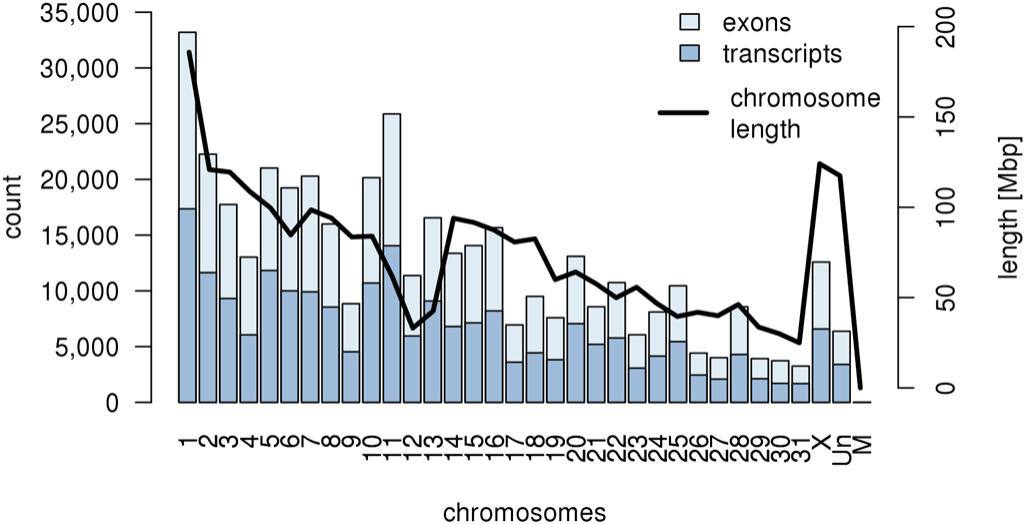
Distribution of exons and transcripts identified in the new transcriptome assembly. The number of exons and transcripts is shown on the y-axis on the left; the length of chromosomes in base pairs is shown on the y-axis on the right.

As expected, we identified the highest number of exons on the biggest chromosome, chromosome 1 (33,197 exons). However, the number of exons did not always correlate with chromosome length. On chromosomes 9 and 10, which have very similar lengths, the numbers of exons differed by more than twofold (8,847 and 20,151 exons). The distribution of the number of exons and number of transcripts per chromosome in the new transcriptome assembly is shown in Fig. 2. The annotation GTF file for the new assembly is available as S1 File.

### Expression quantification

**Fig 3:**
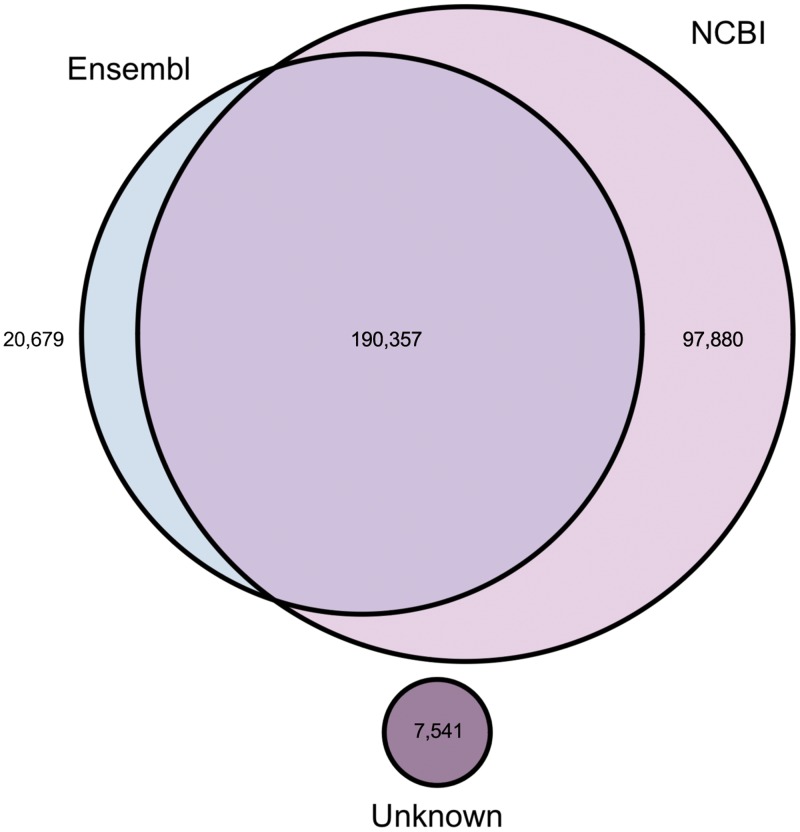
Number of transcripts. The number of transcripts mapped to known/predicted gene models annotated in the Ensembl/NCBI databases and assembled into unknown transcripts.

Most of the read pairs mapped to known/predicted gene models (Ensembl and NCBI). These read pairs were either assembled with complete match to the reference transcripts or predicted to represent new isoforms of the reference genes. Only 1% of the fragments were assembled into new transcripts (5.95 x 10^7^ fragments; 7,541 transcripts). The number of transcripts mapped to Ensembl/NCBI annotated horse genes is shown in Fig. 3.

Transcript expression across samples varied from 0 to 1.62 x 10^6^ FPKM. The calculated median expression per transcript across samples ranged from 0 to 74,180 FPKM. The protein coding reference transcript with the highest expression (max. 26,557 FPKM) was interleukin 8 (*IL8*) that plays a key role in the inflammatory processes attracting leukocytes (mostly neutrophils) from the blood to the sites of inflammation [48]. Among transcripts with the highest expression were also other mediators of inflammation: *CXCL2* (max. 16,806 FPKM), *CCL2* (max. 13,357 FPKM), and *CCL8* (max. 11,441 FPKM). Additional information, such as e.g. transcript length and expression values for each transcript, is given in the supplementary S2 File.

The statistics of the new transcriptome assembly with the number of known and unknown transcripts, after filtering out lowly expressed transcripts (with expression values less than 0.01 FPKM per sample), is shown in Table 1.

**Table 1:** Statistics on the horse PBMCs transcriptome assembly.

Class ^a^	# transcripts	# genes	# single-exon transcripts
Unknown^b^	7,531	6,006	5,281
Complete match to annotated^c^	59,103	26,493	7,705
Novel isoforms^d^	129,309	11,717	0
Other^e^	89,595	12,321	418
TOTAL	285,538	42,602	13,404

^a^The numbers of transcripts identified in the assembly are given after filtering transcripts with expression values less than 0.01 FPKM per sample. The classes are defined according ton the Cufflinks manual [21]:

^b^Unknown, intergenic transcripts

^c^Transcripts with complete match of intron chain to reference transcript

^d^Potentially novel isoform with at least one splice junction shared with a reference transcript

^e^Transcripts with an intron overlapping a reference intron on the opposite strand (n = 74,073); transcripts with generic exonic overlap with a reference transcript (n = 12,700); transcripts with an exonic overlap with reference on the opposite strand (n = 2,780); transcripts falling entirely within a reference intron (n = 39); possible polymerase run-on fragment (n = 2); possible repeat sequence (n = 1).

### Analysis of unknown transcripts

**Fig 4:**
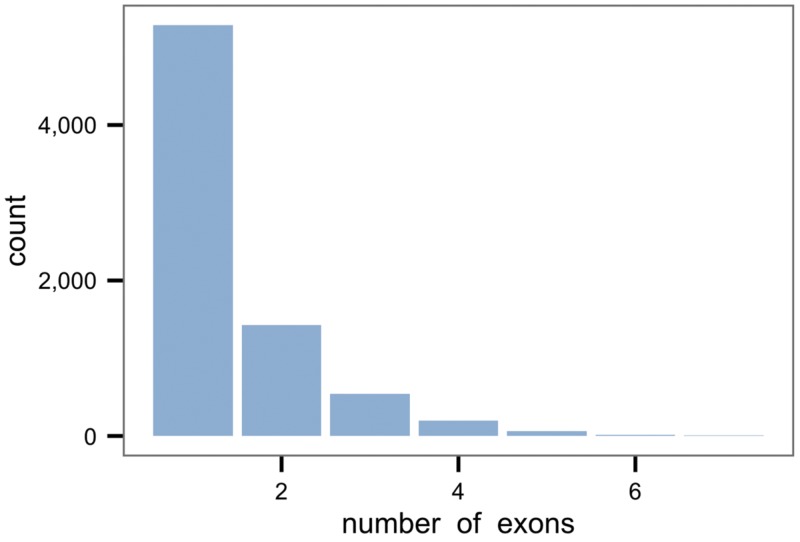
Distribution of the number of exons in the putative new equine transcripts.

The 7,531 unknown transcripts contained between one and seven exons (Fig. 4) and spanned from 49 to 44,080 bp. The majority of these transcripts (n = 5,281; 70%) were single-exon transcripts (Table 1) of length range 49–41,526 bp (median = 1,448 bp). The expression of the unknown transcripts across samples ranged from 0 to 1.87 x 10^6^ FPKM (median = 1.23 FPKM; mean = 389.60 FPKM).

Of the 7,531 unknown transcripts 4,251 (57%) had a hit after blasting against the NCBI EST database, release 130101 (bit score range: 60.2–1,855; median = 268). These transcripts were not considered as horse-specific transcripts as they had a homologous transcript annotated for other species.

### Classification of unknown transcripts

All 7,531 unknown transcripts were subjected to CPC prediction of the coding potential to classify them into protein-coding or non-coding RNA. The majority of the unknown transcripts were classified as non-coding, while 1,104 were predicted to have putative coding potential when both strands were tested (S3 File). Roughly half of these (543 transcripts, 49%) had a coding potential with a score above one.

The median length of proteins, encoded by all unknown transcripts classified as coding, was 126 amino acids (aa) with a range of 16–1,453 aa (S2 Fig.).

**Fig 5:**
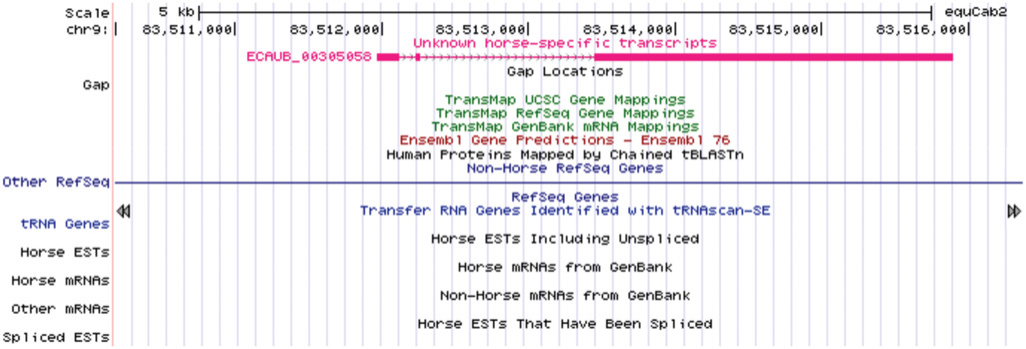
Example of a new putative horse-specific coding transcript. The new transcript is indicated in the UCSC Genome Browser view.

From the potentially coding transcripts 157 did not match any EST deposited in NCBI EST database and were considered to represent new horse-specific transcripts. An example of the potentially new horse-specific coding transcript from chromosome 9 is shown in Fig. 5.

### Validation of potentially coding transcripts

For the validation of the unknown coding transcripts we focused on transcripts with CPC score ≥ 1, at least one spliced intron, an ORF ≥ 300 bp, and mean FPKM values ≥ 1.

**Fig 6:**
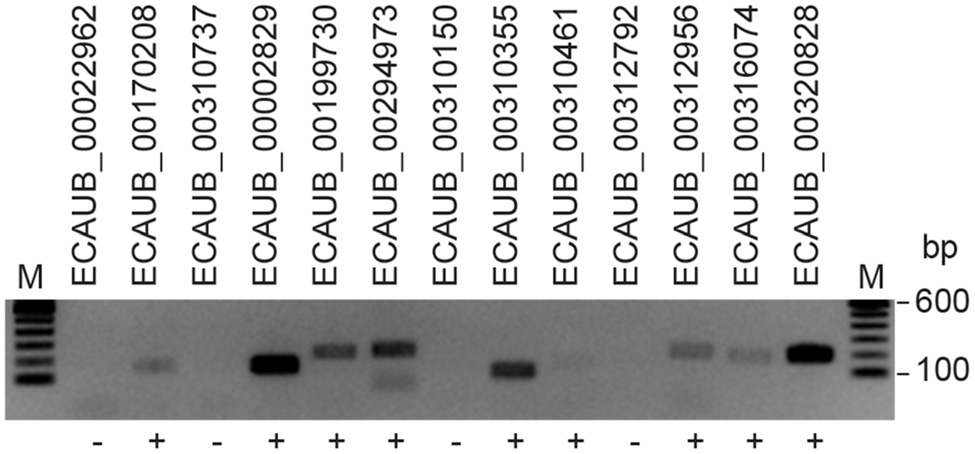
Experimental verification of the expression of predicted new equine transcripts. An agarose gel with transcripts amplified by 36 cycles of RT-PCR is shown.

We selected 13 of these transcripts and designed primer pairs for RT-PCR amplification (S2 Table). Among these 13 transcripts, one was potentially horse-specific (ECAUB_00002829). We selected an RNA sample with predicted high expression, and performed RT-PCR. Nine out of the 13 expected amplification products, including ECAUB_00002829, were detected by electrophoresis (Fig. 6). All nine RT-PCR products were Sanger sequenced and showed a perfect match to the predicted transcripts. The sequences of the verified new horse transcripts are given in S4 File.

## Discussion

The currently available horse genome annotation is still incomplete and contains mostly one single transcript per gene. We used our large RNA-seq dataset to improve the annotation of the horse genome and to characterize the transcriptome of equine PBMCs. Despite high mapping efficiency, less than half of the sequencing reads in our dataset spanned gene regions predicted by the Ensembl pipeline (S1 Fig.).

While the Ensembl pipeline predicts gene models based on known proteins, experimental cDNA and EST sequences [48], the NCBI Gnomon pipeline also makes *ab inito* predictions, which may be fully or partially supported by hypothetical sequence records (with XM_ accessions numbers) from other model organisms, and therefore expands the pool of predicted transcript sequences [49]. We used a merged transcriptome from the Ensembl and NCBI data for our analyses to have the most comprehensive reference transcriptome.

Our equine PBMC transcriptome was expressed from 42,602 predicted genes compared to 63,877 genes annotated in the human genome (Ensembl release 75). However, when comparing these numbers one also needs to consider that we analyzed only PBMCs whereas the human transcriptome annotation is based on a comprehensive collection of tissues and cell types.

The transcript-to-gene ratio in our dataset was 6.70. In contrast, the Ensembl horse annotation contains only slightly more than one transcript per gene (protein coding–1.11; all–1.08; Ensembl release 72). The gene structures reported here were predicted from short-read data and should be taken with some caution. Many of our initially predicted transcripts (n = 30,919; 10%) were expressed at very low level (max. expression across samples < 0.01 FPKM) and filtered out before the secondary analysis steps. There were 2,323 (0.81%) non-overlapping transcripts that belonged to the same gene locus. Only 95 of them represented unknown transcripts. It is possible that those non-overlapping transcripts do not well represent the true transcript structures. Moreover, 5,824 transcripts (2.04%) had the same splice sites and differed only in the length of the first and/or last exon, but were nonetheless described as a new isoform by Cufflinks. We considered this as a discrepancy and these transcripts were marked as duplicates. Sequencing errors, which are common at the ends of the reads, or genomic sequence variations could also be a potential source of such instances. Small changes in the DNA sequence could either mask the true or create novel splice sites with resulting incorrect exon annotations. However, two isoforms with different exon lengths could also both be real. Therefore, we kept all the transcripts identified in our assembly.

We found 7,215 (2.53%) transcripts containing at least one exon with length shorter than 10 nt. Most of these transcripts were associated with Ensembl/NCBI equine transcripts, and only eight represented unknown transcripts. There are only 1,586 (0.75%) exons shorter than 10 nt in the human RefSeq database (NCBI, status from 19^th^ June 2014). These exons belong to 2,241 mRNA sequences from which 1,286 had reviewed status (available sequence data in the literature), 879 validated (after initial review), 65 provisional (not yet subjected for review), 6 predicted, and 5 inferred (predicted and not yet supported by experimental evidence) [50]. Therefore, we suggest treating such transcripts with caution.

Despite the fact that only 4,733 (0.93%) exons did not have a defined strand of origin, we identified 138,559 (27.13%) exons that were reported by Cufflinks on both strands. We will refer to both of these types of exons as ‘unsure’. One explanation for the exons not having strand information is the missing XS tag in the initial BAM file, produced by the GEM mapper. The XS tag gives information on the strand origin of the read and has two values: “+” and “-“. In spliced reads the presence of the XS tag is required by Cufflinks for the correct transcript assembly. In our data, there were 2,838,996 spliced reads (0.01% of the total number of reads) without XS tag. Most of those reads (2,730,237; 96.17%) spanned the unsure exons. We further investigated the reads without XS tags. The size of the skipped region from the reference sequence (“N” in CIGAR string of the SAM format [51]) ranged from to 4 to 499,583 nt and 1,604 reads had skipped regions shorter than 50 nt indicating probably incorrect intron placements. The edit distance from the reference in the reads without XS tag ranged from 1 to 35 bases. In most of those reads (2,449,712; 86.29%) the identified splice site was flanked, at least on one side, by a small deletion, insertion, or soft clipped sequence. Therefore, assignment of the strand of the read origin by the GEM mapper was not possible. From the remaining reads without InDel or clipped sequence, 380,600 reads (13.41%) had more than one skipped region in the CIGAR string. For such short reads (49 bp) more than one skipped region indicated rather incorrect mapping, as it is very unlikely for a transcript to have two exons with the sum of their length less than 49 bp.

Although our assembly is based on short-read data, which may lead to various sorts of artifacts, we strongly believe that our dataset improves the knowledge on the horse transcriptome. We report here many new isoforms of existing genes and new, so far unannotated transcripts, mapping outside of the predicted loci of the horse genome assembly. These unknown transcripts mapping outside previously annotated genes are of major interest to us as they might play a role in determining RAO response and genetic predisposition, and hence were subjected for further analysis and classification. Of the 7,531 unknown transcripts we predicted that 543 have a strong coding potential (above one). Of those, 61 transcripts, expressed from 56 gene loci, did not have a hit after blasting against dbEST (NCBI, release 130101) and were considered as new horse-specific transcripts. The CPC software had a reasonable performance when the horse genome annotation from Ensembl (version 72) was tested. From the 6,218 transcripts known to be expressed in horses (gene type “protein-coding”, transcript status “known”) CPC identified 5,328 (85.69%) as transcripts with coding potential. The CPC software uses both information on open reading frames, and hits against the non-redundant protein database UniProt Reference Clusters (UniRef90) [52]. Therefore the new horse-specific transcripts identified in this study will have lower coding scores since they have no homologs in the protein database. As the coding potential prediction is purely computational, it should be regarded with caution.

From the 13 transcripts used for validation of the unknown transcripts, we successfully amplified 9 transcripts (Fig. 6). Because all transcripts were amplified using the same PCR conditions, it is possible that optimization of the PCR reaction could lead to better amplification of the remaining 4 transcripts. Although many unknown horse-specific transcripts appeared to be well defined, the gene structures from this study should be further investigated and experimentally confirmed.

## Conclusions

Our study provides a significant improvement of the horse transcriptome derived from a large RNA-seq dataset. With 561 samples derived from *in vitro* cultured PBMCs of 85 Warmblood horses we were able to identify roughly 137 thousand transcripts that have not been previously annotated. Moreover, we assembled more than seven thousand putative new horse transcripts from which 61 were potentially new horse-specific transcripts with a strong coding potential. We experimentally confirmed the expression of 9 out of 13 unknown coding transcripts by RT-PCR.

## Supporting Information

S1 Fig The efficiency of mapping with GEM mapper. The boxplots of: *total reads*–the number of sequencing reads per library; *mapped reads*–the number of reads, mapped to the reference genome, per sample; *unique mapping*–the number of reads, mapped uniquely to the reference genome, per sample; *exonic reads*–the number of reads, uniquely mapped to exonic regions of the reference genome, per sample. The percentages show the ratio of total number of reads.
(EPS)

S2 Fig Putative new equine transcripts. Stacked bars representing the distribution of encoded protein length in amino acids. If a transcript had a coding potential on both strands only the strand with the highest score was used for this analysis.
(EPS)

S1 Table Number of replicates per subset of samples studied. (DOCX)

S2 Table The primer sequences for PCR and Sanger sequencing validation of the new transcripts. (DOCX)

S1 File The new horse transcriptome annotation file. Transcripts expressed in *in vitro* stimulated peripheral blood mononuclear cells of RAO-affected and RAO-non-affected thoroughbred horses. Lowly expressed transcripts with max. FPKM per sample < 0.01 were filtered out.
(ZIP)

S2 File Newly assembled transcripts description. For each transcript present in the S1 File the following attributes are given: transcript id, gene id, minimum exon length, maximum exon length, number of exons, strand, chromosome name, start position, end position, length, was the transcript a duplicate, minimum FPKM value per sample, median FPKM value per sample, median FPKM value per sample, class code, nearest reference id, gene short name, reference source.
(ZIP)

S3 File The results of coding potential analysis with Coding Potential Calculator tool. As input transcripts with class code “u” (potentially new transcripts) from S1 File were taken. Both strands (forward and reverse) were tested for coding potential. The file contains header as the first comment line.
(XLSX)

S4 File Sequences of the amplified RT-PCR products in MULTIFASTA format. The consensus sequences of 9 tested unknown horse-specific transcripts were obtained using the Sequencher 5.1 (GeneCodes) software.
(TXT)
