# Supplementary material for: The Transcriptome of Equine Peripheral Blood Mononuclear Cells
Source: PLoS One. 2015 Mar 19;10(3):e0122011. doi: 10.1371/journal.pone.0122011 (PMC4366165; doi:10.1371/journal.pone.0122011)
Supplement: S1 Table — (DOCX) [file pone.0122011.s003.docx]

Supplementary Table S1. Number of replicates per subset of samples studied.

| ***group*** | ***genotype*** | ***stimulation^a^*** | ***condition*** | ***# replicates*** |
| --- | --- | --- | --- | --- |
| 1 | Family 1 | HDE_12 | CTL | 7 |
| 2 | Family 1 | HDE_12 | RAO | 8 |
| 3 | Family 1 | HDE_6 | CTL | 7 |
| 4 | Family 1 | HDE_6 | RAO | 8 |
| 5 | Family 1 | HDE_9 | CTL | 6 |
| 6 | Family 1 | HDE_9 | RAO | 9 |
| 7 | Family 1 | LPS | CTL | 7 |
| 8 | Family 1 | LPS | RAO | 8 |
| 9 | Family 1 | mock | CTL | 6 |
| 10 | Family 1 | mock | RAO | 6 |
| 11 | Family 1 | RCA_1 | CTL | 6 |
| 12 | Family 1 | RCA_1 | RAO | 8 |
| 13 | Family 1 | RCA_4 | CTL | 6 |
| 14 | Family 1 | RCA_4 | RAO | 9 |
| 15 | Family 2 | HDE_12 | CTL | 9 |
| 16 | Family 2 | HDE_12 | RAO | 8 |
| 17 | Family 2 | HDE_6 | CTL | 9 |
| 18 | Family 2 | HDE_6 | RAO | 7 |
| 19 | Family 2 | HDE_9 | CTL | 9 |
| 20 | Family 2 | HDE_9 | RAO | 7 |
| 21 | Family 2 | LPS | CTL | 9 |
| 22 | Family 2 | LPS | RAO | 8 |
| 23 | Family 2 | mock | CTL | 9 |
| 24 | Family 2 | mock | RAO | 6 |
| 25 | Family 2 | RCA_1 | CTL | 9 |
| 26 | Family 2 | RCA_1 | RAO | 8 |
| 27 | Family 2 | RCA_4 | CTL | 8 |
| 28 | Family 2 | RCA_4 | RAO | 7 |
| 29 | Unrelated | HDE_12 | CTL | 28 |
| 30 | Unrelated | HDE_12 | RAO | 23 |
| 21 | Unrelated | HDE_6 | CTL | 28 |
| 32 | Unrelated | HDE_6 | RAO | 22 |
| 33 | Unrelated | HDE_9 | CTL | 28 |
| 34 | Unrelated | HDE_9 | RAO | 22 |
| 35 | Unrelated | LPS | CTL | 29 |
| 36 | Unrelated | LPS | RAO | 23 |
| 37 | Unrelated | mock | CTL | 26 |
| 38 | Unrelated | mock | RAO | 23 |
| 39 | Unrelated | RCA_1 | CTL | 26 |
| 40 | Unrelated | RCA_1 | RAO | 23 |
| 41 | Unrelated | RCA_4 | CTL | 24 |
| 42 | Unrelated | RCA_4 | RAO | 22 |

^a^ HDE_12: hay dust extract at 12 µg/ml;

^b^ HDE_9: hay dust extract at 9 µg/ml;

^c^ HDE_6: hay dust extract at 6 µg/ml;

^d^ RCA_4: hay dust extract at 4 µg/ml;

^e^ RCA_1: hay dust extract at 1 µg/ml;

^f^ LPS: lipopolysaccharides at 250 ng/ml;

^g^ mock: no stimulating factor
