# Supplementary material for: The Transcriptome of Equine Peripheral Blood Mononuclear Cells
Source: PLoS One. 2015 Mar 19;10(3):e0122011. doi: 10.1371/journal.pone.0122011 (PMC4366165; doi:10.1371/journal.pone.0122011)
Supplement: S2 Table — (DOCX) [file pone.0122011.s004.docx]

Supplementary Table S7. The primer sequences for PCR and Sanger sequencing validation of the new transcripts.

| NR | PRIMER | START | LENGTH | TM | GC% | ANY_TH | 3'_TH | HAIRPIN | SEQ |
| --- | --- | --- | --- | --- | --- | --- | --- | --- | --- |
| 1 | ECAUB_00022962_L | 184 | 20 | 58 | 50 | 2 | 0 | 0 | CACATTTCTCACTGTGGGCT |
| 1 | ECAUB_00022962_R | 420 | 20 | 59 | 55 | 0 | 0 | 0 | AGCTATGACACCAGTGACCC |
| 2 | ECAUB_00170208_L | 8385 | 20 | 59 | 55 | 0 | 0 | 0 | AGAATGCTCTAAGGCCCTGG |
| 2 | ECAUB_00170208_R | 8539 | 20 | 59 | 55 | 0 | 0 | 0 | AGTCTCTAGCCTGCCCAAAG |
| 3 | ECAUB_00310737_L | 582 | 22 | 58 | 41 | 0 | 0 | 0 | GGCCACAAATGCCATCAATTAT |
| 3 | ECAUB_00310737_R | 872 | 20 | 59 | 55 | 0 | 0 | 0 | CCACCAGGCAGAAAACGATG |
| 4 | ECAUB_00002829_L | 2088 | 20 | 59 | 60 | 0 | 0 | 0 | GTCTCATCTGGGCTACTGGG |
| 4 | ECAUB_00002829_R | 2238 | 20 | 59 | 55 | 23 | 0 | 0 | GAAGGGACGCTTCATCTCCT |
| 5 | ECAUB_00199730_L | 2297 | 22 | 58 | 45 | 2 | 0 | 0 | GGCTTTAGTTCCAGAAAGTCCA |
| 5 | ECAUB_00199730_R | 2525 | 21 | 59 | 52 | 0 | 0 | 0 | TCCCTATCTGATCCCTCCAGA |
| 6 | ECAUB_00294973_L | 362 | 20 | 59 | 50 | 0 | 0 | 0 | AAGTGGATGTGCAAAGGTGG |
| 6 | ECAUB_00294973_R | 596 | 20 | 59 | 50 | 7 | 7 | 0 | CTTGAAGAAAAGCTGGGCCA |
| 7 | ECAUB_00310150_L | 1123 | 22 | 59 | 50 | 0 | 0 | 0 | ACCTATATGAGAAAGCCCCTGG |
| 7 | ECAUB_00310150_R | 1329 | 20 | 59 | 50 | 15 | 0 | 0 | AACACCTGCAGCCTGACTAT |
| 8 | ECAUB_00310355_L | 328 | 18 | 59 | 67 | 1 | 0 | 0 | GGGACAGGACTCCTTGGG |
| 8 | ECAUB_00310355_R | 427 | 19 | 59 | 63 | 0 | 0 | 0 | GCTGAGGTCTCCGAGGAAG |
| 9 | ECAUB_00310461_L | 2932 | 20 | 59 | 55 | 0 | 0 | 0 | ATGCTGGAGGTGTATGAGGG |
| 9 | ECAUB_00310461_R | 3081 | 20 | 60 | 60 | 9 | 4 | 0 | GGTCTGCCTGGCTCTAGAAC |
| 10 | ECAUB_00312792_L | 1194 | 21 | 59 | 48 | 0 | 0 | 0 | GCTGACGCCTCATTACAAAGT |
| 10 | ECAUB_00312792_R | 1391 | 20 | 59 | 55 | 0 | 0 | 0 | CACACTTCACTCACACTGCC |
| 11 | ECAUB_00312956_L | 2744 | 20 | 59 | 55 | 0 | 0 | 0 | CTTGAATCTGAGCCCCTGGA |
| 11 | ECAUB_00312956_R | 2951 | 20 | 59 | 50 | 0 | 0 | 0 | GCAGCCATTTGTGCTTCTGA |
| 12 | ECAUB_00316074_L* | 575 | 21 | 58 | 48 | 0 | 0 | 0 | ACCACAATCTTACTGTCTCGC |
| 12 | ECAUB_00316074_R* | 750 | 20 | 59 | 55 | 5 | 5 | 0 | TCAGACTAGAGGGCCAGAGT |
| 13 | ECAUB_00320828_L | 569 | 21 | 58 | 48 | 0 | 0 | 0 | GCGAGACAGTAAGATTGTGGT |
| 13 | ECAUB_00320828_R | 758 | 20 | 59 | 55 | 0 | 0 | 0 | CAGGAACATGTGTAGGCAGC |

* This pair of primers was not specific. The product could have been derived from both ECAUB_00316074 and ECAUB_00320828. These transcripts are on opposite strands and both represent unknown transcripts.
